# Embracing New Love: Why Customers Are Loyal to Plant Extract‐Based Skin‐Care Cosmetics

**DOI:** 10.1111/jocd.16731

**Published:** 2025-01-03

**Authors:** I‐Hsuan Wu, Chaoyun Liang

**Affiliations:** ^1^ Department of Bio‐Industry Communication and Development National Taiwan University Taipei Taiwan; ^2^ Division of Quality Compliance and Management Food and Drug Administration Taipei Taiwan

**Keywords:** consumer loyalty, involvement, perceived value, plant extract‐based skin‐care products, prior experience

## Abstract

**Background:**

In recent years, increases in consumer awareness regarding health and the environment have enhanced their willingness to purchase plant extract‐based skin‐care products. Although the skin‐care product industry has paid increasing attention to consumer behavior in recent years, few studies have investigated customer loyalty to this type of product; in‐depth research is urgently required to fill this gap.

**Aims:**

This study investigated Taiwanese skin‐care products derived from plant extracts by identifying the relationships between consumer prior experience, involvement, perceived value, and loyalty. It also examined how demographic characteristics influence consumer loyalty.

**Patients/Methods:**

An online survey yielded 920 valid samples for statistical analysis. Three constructs of involvement, namely product, message, and situational involvement; three constructs of perceived value, namely functional, emotional, and social value; and three constructs of consumer loyalty, namely repurchase intention, willingness to receive information, and willingness to pay, were identified through factor analysis.

**Results:**

The respondents' perceived value significantly influenced their loyalty toward Taiwanese plant‐extract‐based skin‐care products. The factor with the strongest effect on repurchase intention was emotional value, followed by functional value, product involvement, and social value. The factor with the strongest influence on willingness to receive information and willingness to pay was functional value, followed by product involvement, situational involvement, and emotional value.

**Conclusions:**

Respondents with higher educational levels were less likely to repurchase and less willing to receive information on plant extract‐based skin‐care products. Moreover, men were more willing to pay price premiums for these products than were women.

## 1 Introduction

People often struggle with skin conditions such as a dull complexion, enlarged pores, skin aging, and the proliferation of pimples and acne. Various types of skin‐care products offer different functions, such as whitening, antiaging, antiacne, moisturizing, antisensitivity, and sun protection [1]. However, conventional skin‐care products contain a variety of chemical ingredients, which can potentially irritate the skin, accelerate aging, and even induce tumors. Consequently, consumers have begun to prefer natural ingredients [2, 3]. Natural ingredients are extracted directly from animals and plants. The sources of natural ingredients include herbs, flowers, leaves, fruits, livestock skins, fats, and shells [4]. Most of the natural ingredients added to skin‐care products are plant extracts. These extracts include antioxidants, tyrosinase inhibitors, and antibacterial agents with antiaging, whitening, antibacterial, and other effects [5, 6]. The effectiveness of plant extract‐based skin‐care (PEBSC) products depends on the skin's ability to absorb their ingredients and on the physiological effects of these ingredients. The extraction method, the ratio of plants to solvents, and the content of active ingredients can influence product effectiveness [7].

The skin‐care product market has substantial growth potential. Despite the decline in the growth rate of the overall cosmetics market since the outbreak of the COVID‐19 pandemic, the skin‐care product market has grown during this time [8, 9]. However, Taiwan's skin‐care product industry is overly reliant on imported raw materials, the supply of which is controlled by major international manufacturers [10]. Because of the global instability arising from various factors (such as the Russo‐Ukrainian war, which has disrupted agricultural production, and the COVID‐19 pandemic, which resulted in closures of transportation routes), the skin‐care product industry is facing a shortage of raw materials, which has resulted in increasing costs and ultimately affected consumer behavior. This shortage of raw materials must be overcome urgently by identifying appropriate local agricultural products to achieve supply independence. Research and development regarding key raw materials can not only fill the gap in industrial demand but also increase the competitiveness of domestic skin‐care products in the international market [11]. Although Taiwan has a thriving agricultural industry, agricultural by‐products and waste can cause environmental pollution. However, agricultural by‐products are rich in carbohydrates, proteins, lipids, and other compounds that can be used as raw materials for skin‐care products, health products, medicines, and other products [6, 12]. Skin‐care product manufacturing requires little investment, has low risk, and can provide high gross profits. Therefore, the Taiwanese government should invest resources in researching the efficacy of PEBSC products and assist agricultural enterprises in developing these products.

In recent years, increases in consumer awareness regarding health and the environment have enhanced their willingness to purchase PEBSC products. Consumer lifestyles and personal experiences affect their loyalty to skin‐care products [13, 14]. Although positive consumption experiences promote customer loyalty, poor experiences that do not meet expectations can result in negative consumer attitudes and low repurchase intention. Involvement is closely related to prior experience. The levels of consumer involvement with a specific product, the product information, and the consumption situation can differ and are affected by their needs or interests. A higher degree of involvement suggests greater attention paid to consumption and greater customer loyalty [15]. Positive experiences and in‐depth involvement are closely related to a high degree of perceived value. Perceived value refers to a consumer's comprehensive evaluation of a product on the basis of product utility and loss. A higher evaluation naturally strengthens customer loyalty [16]. This behavioral pattern is also reflected in the consumption of skin‐care products [17, 18]. In addition, demographic characteristics, especially sex, age, income, place of residence, and skin type can strongly affect customer loyalty for skin‐care products [19, 20]. Although the skin‐care product industry has paid increasing attention to consumer behavior in recent years, few studies have investigated customer loyalty to PEBSC products; in‐depth research is urgently required to fill this gap [11, 21].

PEBSC products have high consumer appeal and favorable global market prospects [22]. Therefore, in the present study, Taiwanese PEBSC products were selected as the research target, and the effects of prior experience, involvement, and perceived value on customer loyalty were investigated. Moreover, the effects of various demographic factors on customer loyalty were determined. The goal of the aforementioned investigations was to better understand consumption patterns for PEBSC products in Taiwan; perform theoretical innovation; provide recommendations regarding research and design, production, and marketing for Taiwan's agricultural enterprises; and offer policy suggestions to the Taiwanese government to assist it in achieving improvements in the skin‐care and agricultural industries.

## 2 Literature Review

### 2.1 Market Trends of PEBSC Products

Skin‐care products are designed to promote skin health and renewal and are typically used after skin cleansing. They contain a variety of active ingredients, such as *aloe vera* , cabbage, niacinamide, titanium dioxide, and hyaluronic acid [23, 24]. These products, especially those derived from plant extracts, have become increasingly popular among consumers [25]. Plant extracts are compounds separated from various parts of plants through physical or chemical extraction methods. However, the composition and content of these extracts can vary considerably because of factors such as climate, soil composition, latitude, season, harvest time, and field management. This variability presents a challenge in standardizing the manufacturing process of skin‐care products [26].

Surplus agricultural production is common in Taiwan. Researchers can examine these surplus products and identify their functional ingredients that can be used for the creation of skin‐care products; this approach can increase resource use efficiency and farmer income [11]. Using local agricultural products to create skin‐care products has several benefits, including shortened transportation distances and reduced CO_2_ emissions. This approach also promotes environmental protection, local heritage, job creation, and local agricultural development [27]. Domestic operators can also incorporate Taiwanese culture, history, and emotions as an added value to enhance Taiwan‐made skin‐care products to attract consumers [28]. In recent years, cosmetic companies have begun to experiment with natural and environmentally friendly raw materials in response to growing public concern for environmental sustainability [29]. With the improvement of living standards and technological advancement, products containing plant extracts have become the preferred choice for many skin‐care product consumers [30].

Despite the impact of the COVID‐19 pandemic, the cosmetics industry remains one of the fastest‐growing and most profitable sectors in the global economy. Although the growth rate of the global cosmetics market decreased by 8% in 2020, that of skin care and beauty products grew by 2%. As the pandemic subsides, the market is expected to rise at a compound annual growth rate of 4.6% from 2022 to 2030 [31]. The rising awareness of health and environmental protection, coupled with the booming market for natural and organic skin‐care products, has made PEBSC products an emerging focus for consumers. Consumers believe that these products are safe, are effective, have no side effects, have high value for money, and have social significance.

### 2.2 Consumer Loyalty and Perceived Value

The success of a business often hinges on maintaining good customer relationships because acquiring new customers is often more challenging than retaining existing ones [32]. Customer loyalty encompasses attitudinal and behavioral aspects. Attitudinal loyalty refers to a customer's psychological preferences, such as intent to repurchase, whereas behavioral loyalty refers to actual purchasing behavior, such as the number and frequency of purchases [33]. Oliver [34] proposed a customer loyalty model with four levels. First, customers rationally form a preference for a particular good or service (cognitive loyalty), after which they develop a preference for the product on the basis of prior purchasing experience (emotional loyalty) and develop an emotional commitment to repurchase the product (intention loyalty). Finally, they develop an intention to repurchase the product repeatedly even in the presence of conflicting incentives (action loyalty). Loyal customers are characterized by high consumption, frequent repeat purchases, low price sensitivity, willingness to recommend products, preference for the original products despite competition, and willingness to maintain a relationship with the company by purchasing other products and receiving marketing information [35, 36].

Perceived value is a customer's overall assessment of the utility of a product or service; this assessment is based on their perception of the benefits that they receive and the costs that they incur [37]. Perceived value has three characteristics: (1) it is diverse, encompassing various perceived benefits and costs; (2) it is subjective, with different customers having varied perceptions of the same product; and (3) it is competitive, with companies that deliver superior value gain achieving sustainable competitive advantages [38]. Sheth, Newman, and Gross [39] proposed consumption value theory with five categories of perceived value: functional, social, emotional, epistemic, and conditional value. Functional value refers to the quantifiable or unquantifiable price, function, use, and quality of the product or service itself; social value refers to connections with other social groups (obtaining group recognition, complying with social norms, gaining a sense of belonging, and demonstrating internal image or display of status); emotional value refers to consumers' emotional reactions to products or services; epistemic value refers to the novelty of a product or service that can arouse customers' curiosity and desire for knowledge; and conditional value refers to the higher functional or social value that consumers can temporarily obtain from a product in specific situations [40]. Sweeney and Soutar [41] suggested that these value dimensions are not independent, and the relative importance of each value might differ between products or services. Studies on skin‐care products have consistently measured perceived value using these dimensions (e.g., [42]).

Perceived value is a key determinant of customer loyalty. A higher perceived value leads to a stronger purchase intention and more robust customer loyalty [43]. Perceived value can influence customer loyalty in various ways. For instance, Ghazali et al. [44] found that higher perceived health, safety, hedonic value, and social value increased repurchase intention for organic cosmetics, Song, Guo, and Zhang [45] found that social value, price, and brand value positively affect customer loyalty to environmentally friendly cosmetics. Choi and Lee [46] indicated that the factors determining willingness to repurchase environmentally friendly cosmetics are often related to identity, acceptance, and pride, which are aspects of social value. Therefore, the following hypothesis is proposed with regard to Taiwan‐made, PEBSC products:

*Perceived value positively affects customer loyalty*.

### 2.3 Prior Experience, Perceived Value, and Customer Loyalty

Prior experience refers to customer reactions and feedback after exposure to an environment, a product, or a service; experiences include consumer cognition, preferences, emotions, beliefs, physiological and psychological reactions, and sources of achievement [47]. Prior experience encompasses personal experiences, lifestyle, and interactions with the product [48]. Taiwan's dynamic climate can significantly affect consumer experiences and perceived benefits of using skin‐care products, which is reflected in consumer behavior. For example, in Taiwan's summer, sun exposure may cause solar dermatitis, solar keratosis, cell damage, and accelerated skin aging. Varying weather and high‐temperature differences between day and night might induce urticaria, and extreme weather events may double the chance of bacterial skin infection. Summer humidity and heat can cause sweat rash, impetigo, and fungal infection and exacerbate contact dermatitis. The dry, cold winter can cause dry skin [49, 50, 51]. People who regularly engage in outdoor activities prioritize skin‐care products that offer ultraviolet protection.

The consumption experience is a crucial factor in evaluations of perceived value [52]. As the quality of experience increases, so does the customer's perceived value. Customers whose expectations are met by consumption tend to have a positive feeling toward the relevant product, have a strong emotional connection with the product, have a high evaluation of the product, and continue using the product [53]. Prior experiences can influence customer impressions of products and services, further solidifying their loyalty [52]. Several studies have indicated that perceived value can act as a mediating variable between consumption experience and purchase intention [54, 55]. Therefore, the following research hypotheses are proposed:

*Prior experiences positively affect perceived value*.

*Prior experiences positively affect customer loyalty*.

*Perceived value enhances the effect of prior experience on customer loyalty*.

### 2.4 Involvement, Perceived Value, and Customer Loyalty

Social judgment theory is based on self‐persuasion and posits that people perceive and evaluate new information on the basis of their existing attitudes. This theory led to the development of involvement theory [56]. Involvement refers to an individual's interest in and concern for a specific thing or the psychological state triggered by specific stimuli and situations. It represents an individual's perception regarding the relevance of something, which depends on their needs, interests, and values [57]. Consumers with high involvement resist accepting opinions that contradict their original ideas [15]. Initially used in media research, involvement theory is now widely applied in marketing [58]. Consumers with high involvement actively and extensively collect information, whereas those with low involvement are passive, expose themselves to limited information, and perform decision‐making with the simple stages of awareness, trial, and adoption [15].

Previously, scholars categorized involvement as enduring, situational, and response involvement [59]. Enduring involvement is an individual's ongoing concern for things according to their needs, interests, and values, and such involvement is unaffected by external changes. Situational involvement is a temporary state triggered by a specific situation, which reverts to the original state once a goal is achieved or the situation changes. Response involvement refers to the behavioral and cognitive outcomes resulting from the combination of situational and enduring involvement [60, 61]. Involvement was later reclassified into product, message, and situation involvement [58]. Product involvement is similar to enduring involvement but is more closely tied to consumer products. Message involvement relates to an individual's concern for product information or their psychological state when they are exposed to this information. Situational involvement refers to personal cognition and the attention that an individual pays to consumption behavior in a purchasing situation [15]. The present study adopted the later classification because of its clear distinction between concepts and its consideration of message impact.

Studies have indicated that product involvement significantly influences perceived value and customer loyalty. Consumers' perceived value varies depending on their level of product involvement, which also enhances customer loyalty [62]. When consumers with high product involvement are satisfied, they exhibit high customer loyalty, which indicates that perceived value mediates the relationship between product involvement and customer loyalty. Similarly, message involvement positively affects perceived value and customer loyalty; if the message content is engaging, it can stimulate consumer recall, understanding, be more persuasive, reduce perceived sacrifice, and improve perceived value [63]. Situational involvement also affects perceived value and customer loyalty, particularly when time is limited and perceived risk is reduced [61]. Customers with firm loyalty insist on purchasing a specific brand even if they urgently need the relevant product or the store is out of stock. Wu, Liang, and Ip [15] claimed that situational involvement affects purchasing decisions to a greater extent than product or message involvement. Perceived value also mediates the effect of situational involvement on customer loyalty [64]. On the basis of these findings, the following research hypotheses are proposed:

*Customer involvement positively affects perceived value*.

*Customer involvement positively affects customer loyalty*.

*Perceived value enhances the effect of involvement on customer loyalty*.

### 2.5 Effects of Various Demographic Characteristics on Customer Loyalty

The demographic characteristics examined in this study were sex, age, place of residence, educational level, monthly disposable income, and skin type [65]. Consumption behavior for skin‐care products varies between men and women. Women prefer products with diverse features and are generally less loyal than men. Men repurchase products primarily because of satisfaction with the product's function, whereas women value the interaction with the salesperson during purchase [66]. Men's spending on skin‐care products has been increasing annually, thereby expanding the market [67]. Men are more loyal to high‐priced products than women and associate these products with stable quality, low risk, and high status [68]. Women use a wider variety of skin‐care products, prefer lotions and lip balms, and are more loyal to specific brands [69]. Matić and Puh stated that women care more about function and emotion and have stronger repurchase intention for natural skin‐care products than men [70].

Customer age also influences skin‐care product consumption behavior. Older consumers have extensive shopping experience and make decisions according to this experience and safety. By contrast, younger consumers consider satisfaction but might prioritize sales staff information, enjoy change, and value openness [66]. Older consumers are generally more loyal than younger ones. Older consumers, who typically have simpler lifestyles and higher disposable income, have higher demand and loyalty for locally sourced natural skin‐care products [71].

Place of residence, which is an external factor, also affects skin‐care product purchasing decisions. Because of urban–rural differences in income and price levels, customer loyalty varies between urban and rural areas [72]. Lin and Chuang [73] indicated that people in southern Taiwan are more pragmatic and conservative compared with those in northern Taiwan, whereas people in northern skin‐care product manufacturers often segment the market on the basis of regional characteristics to develop different sales strategies, thereby increasing customer satisfaction and loyalty [74].

Education level also affects customer loyalty, albeit to a lesser extent than other demographic variables. Evanschitzky and Wunderlich [66] found that lower education levels were associated with contextual factors having a stronger effect on loyalty. People with higher education levels tend to collect and process more information and make more cautious consumption decisions [73]. Compared with people with a low education level, highly educated people, who are better at collecting and understanding product information, are expected to have higher repurchase intentions for local PEBSC products, which they recognize as being rich in bioactive substances [8].

Customer loyalty can be influenced by monthly disposable income. Consumers with high disposable income tend to prioritize product quality and after‐sales service but are resistant to sales, promotions, and discount products, which results in higher customer loyalty. Conversely, those with lower disposable income are more likely to be attracted to lower‐priced alternatives, which makes it easier for them to switch products and thus reduces their loyalty [75].

Customer loyalty to specific skin‐care products can also be affected by their skin type and condition. The sebum on the skin surface, which regulates skin humidity and protects the skin, can affect skin quality [76]. However, individual skin conditions can vary because of factors such as innate physique, acquired care, nature of work, entertainment orientation, and product attribute requirements. Skin types can be categorized into oily, dry, combination, and normal, and no single skin‐care product can cater to all skin types simultaneously. Different skin types require different treatments and products. Consumers' expectations for skin‐care products vary, including in aspects such as tolerance, safety of use, and pleasure [77, 78]. Therefore, the following hypothesis is proposed:

*For consumers of Taiwanese PEBSC products, customer loyalty is influenced by factors such as sex, age, place of residence, educational level, monthly disposable income, and skin type*.

**FIGURE 1:**
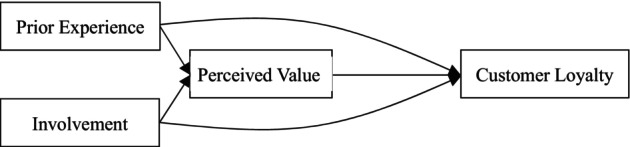
Developed research model for consumers of Taiwanese PEBSC products.

The research model developed in this study is displayed in Figure 1.

## 3 Methods

Taiwanese PEBSC products are uniquely important from agricultural and economic development perspectives [79, 80]. The present study focused on Taiwanese consumers aged over 18 years with experience in using domestic PEBSC products. Of 965 respondents, those with an item variation of below 0.2 were excluded; thus, 920 valid cases remained for further analysis. This study's questionnaire is divided into five sections, namely those related to prior experience, involvement, perceived value, customer loyalty, and demographic characteristics. It comprises 37 items, which are detailed as follows:

1. Prior experience: Two questions were developed with reference to three documents including Hsu et al. [50], Theopilus et al. [48], and Zandman‐Goddard et al. [51].
2. Involvement: Seventeen questions are developed based on the four literatures including Huang [60], Im and Ha [61], Wu, Liang, and Ip [15], and Zaichkowsky [57].
3. Perceived value: Twelve questions are developed based on four literatures including Kung, Wang, and Liang [40], Sheth, Newman, and Gross [39], Sweeney and Soutar [41], and Zeithaml [37]
4. Customer loyalty: Six questions are developed with reference to three documents including Narayandas [35], Srivastava and Rai [36], and Venkateswarlu, Ranga, and Sreedhar [32].
5. Demographic characteristics: Six questions are developed including sex, age, place of residence, educational level, monthly disposable income, and skin type.

The Likert scale is a common attitude measurement tool in social sciences and was used in this study. In accordance with the recommendations of Chomeya [81] and Taherdoost [82], a 6‐point Likert scale was used in this study to ensure that respondents could clearly express their opinions. The scale endpoints ranged from 1 (*strongly disagree*) to 6 (*strongly agree*). The purpose of the study was clearly communicated to the respondents at the beginning of the questionnaire, and the respondents were explicitly told that the survey was anonymous and that their privacy was protected. This study was conducted on the SurveyCake online platform. The QR code linking to the questionnaire was shared on forums and online groups related skin‐care products. The survey was conducted from July 1 to August 15, 2023. All questionnaire items were single‐choice questions, and no item could be left blank, thereby ensuring that no values were missing.

Subsequently, confirmatory factor analysis (CFA) was conducted using Amos 25.0 to assess the factor structure of the items. Factor loadings, means, standard deviations, composite reliabilities (CRs), and average variance extracted (AVE) values were obtained. A structural model was then evaluated using the maximum likelihood method. The heterotrait–monotrait (HTMT) ratio of correlations was used to evaluate the discriminant validity between constructs. The model's goodness of fit with the hypotheses was tested in terms of χ^2^, root mean square error of approximation (RMSEA), standardized root mean squared residual (SRMR), comparative fit index (CFI), and Tucker–Lewis index (TLI) [83]. Furthermore, standardized beta values were used to determine whether the hypotheses were supported.

## 4 Results

### 4.1 Descriptive Analysis

The descriptive statistics are reported in Table 1.

**TABLE 1:** Respondent demographics (*N* = 920).

Demographic variables	Percentage (Frequency)				
Sex	Men		Women		
	36.3% (334)		63.7% (586)		
Age	≤ 30		31–40	≥ 41	
	18.7% (172)		35.4% (326)	45.9% (422)	
Place of residence	Northern Taiwan		Non‐northern Taiwan		
	50.8% (467)		49.2% (453)		
Educational level	High school and under		Undergraduate	Postgraduate	
	12.6% (116)		59.4% (546)	28.0% (258)	
Monthly disposable income	≤ 320 USD	321–950 USD	951–1600 USD	≥ 1601 USD	
	22.9% (211)	37.1% (341)	23.0% (212)	17.0% (156)	
Skin type	Dry	Normal	Oily	Combination	Unsure
	6.8% (63)	15.0% (138)	27.3% (251)	45.8% (421)	5.1% (47)

*Note:* Monthly disposable income refers to the average monthly income remaining after the deduction of monthly fixed expenses.

### 4.2 Confirmatory Factor Analysis

In this study, maximum likelihood estimation was performed in CFA to determine the factor structures of involvement, perceived value, and customer loyalty. The analysis indicated that involvement can be divided into three factors, namely product, message, and situational involvement; perceived value can be distinguished into three factors, namely functional, emotional, and social value; and consumer loyalty can also be divided into three factors, namely repurchase intention, willingness to receive information, and willingness to pay. The results (*χ* ^2^ = 1845.503, df = 584, χ^2^/df = 3.16, *p* < 0.001, RMSEA = 0.05, SRMR = 0.04, CFI = 0.93, and TLI = 0.92) indicate a satisfactory fit in accordance with the criteria suggested by Hu and Bentler [84]. The factor loadings of the items ranged from 0.53 to 0.88, the AVE values of the constructs ranged from 0.45 to 0.75, and the CRs of the constructs ranged from 0.61 to 0.89; these results indicated that all constructs had acceptable convergent validity (Table 2). In Table 2, “these products” refer to Taiwanese PEBSC products.

**TABLE 2:** Results of CFA (*N =* 920).

Construct/Item	FLs	*M*	SD	CR	AVE
**Prior experience**				0.61	0.45
I have experienced allergies from using skin‐care products	0.74	4.73	1.13		
I have used skin‐care products that did not achieve the results that I expected	0.60	3.59	1.45		
**Product involvement**				0.89	0.49
These products are gentle and nonirritating	0.76	4.59	0.88		
These products can moisturize	0.75	4.46	0.83		
These products can beautify the skin	0.72	4.19	0.87		
These products are suitable for sensitive skin	0.73	4.36	0.93		
These products can fight aging	0.70	4.00	0.91		
I love the natural scent of these products	0.66	4.72	0.91		
These products can fight acne	0.65	3.91	0.94		
These products are generally of good quality	0.63	4.16	0.89		
**Message involvement**				0.84	0.51
I pay attention to the claims of the products regarding skin types	0.80	4.98	0.87		
I read the instructions on the packaging of the products	0.79	5.02	0.91		
I pay attention to the functional claims of each product	0.73	4.85	0.92		
I pay attention to the source of ingredients stated on the packaging of the products	0.69	4.92	1.00		
I pay attention to the price information of these products.	0.53	5.07	0.95		
**Situational involvement**				0.75	0.45
I pay attention to the design quality of the product packaging when purchasing these products	0.75	4.33	0.96		
I pay attention to the unique style of store decoration while purchasing these products	0.67	3.67	1.13		
I pay attention to the usability of shopping websites when purchasing these products	0.65	4.57	0.98		
I like persuasive celebrity endorsements when purchasing these products	0.53	3.64	1.15		
**Functional value**				0.85	0.52
The variety of these products satisfies my desire to try new things	0.78	4.12	0.99		
The claimed functions of these products amaze me	0.74	4.17	0.98		
Using these products benefits me at work	0.72	3.74	1.04		
I use these products to increase my personal charm	0.69	3.95	1.02		
I love trying out combinations of these products	0.68	3.96	1.07		
**Emotional value**				0.86	0.61
I feel comfortable with these products	0.81	4.33	0.83		
I have high confidence in these products	0.81	4.21	0.84		
Using these products makes me happy	0.78	4.34	0.85		
The research and development process for these products is diligent	0.73	4.50	0.84		
**Social value**				0.85	0.66
Purchasing these products means doing your best to care for the Earth	0.88	4.32	1.08		
Purchasing these products means supporting local agriculture	0.79	4.41	1.04		
Purchasing these products as gifts can give people a good impression of being environmentally friendly	0.76	4.25	1.05		
**Repurchase intention**				0.86	0.75
I will continue to purchase these products	0.88	4.21	0.91		
I will prioritize purchasing these products	0.85	4.24	0.91		
**Willingness to receive information**				0.76	0.61
I am happy to receive information from the manufacturer of these products	0.80	3.82	1.12		
I am happy to receive information about these products from my relatives and friends	0.77	3.99	1.05		
**Willingness to pay**				0.67	0.50
I hope to purchase all of these products at once	0.73	3.78	1.09		
Even if the price of these products increases, I am still willing to buy them	0.68	3.71	1.04		

*Note:* “These products” refer to Taiwanese PEBSC products.

Abbreviations: AVE, average variance extracted; CR, composite reliability; FL, factor loading; M, mean; SD, standard deviation.

### 4.3 HTMT Ratio of Correlations

The obtained HTMT ratios are presented in Table 3. The recommended HTMT ratio threshold of 0.9 was used to obtain sufficient evidence of discriminant validity [85].

**TABLE 3:** HTMT of the constructs (*N* = 920).

Construct	1	2	3	4	5	6	7	8	9	10
Prior experience (1)	(0.67)									
Product involvement (2)	0.23	(0.70)								
Message involvement (3)	0.38	0.50	(0.71)							
Situational involvement (4)	0.15	0.41	0.35	(0.66)						
Functional value (5)	0.23	0.71	0.45	0.57	(0.72)					
Emotional value (6)	0.24	0.79	0.54	0.49	0.81	(0.78)				
Social value (7)	0.22	0.64	0.43	0.43	0.73	0.73	(0.81)			
Repurchase intention (8)	0.21	0.63	0.43	0.41	0.76	0.79	0.67	(0.87)		
Willingness to receive information (9)	0.20	50	0.35	0.42	0.68	0.58	0.55	0.59	(0.78)	
Willingness to pay (10)	0.13	0.56	0.29	0.52	0.70	0.69	0.64	0.84	0.73	(0.71)

### 4.4 Structural Equation Modeling

**FIGURE 2:**
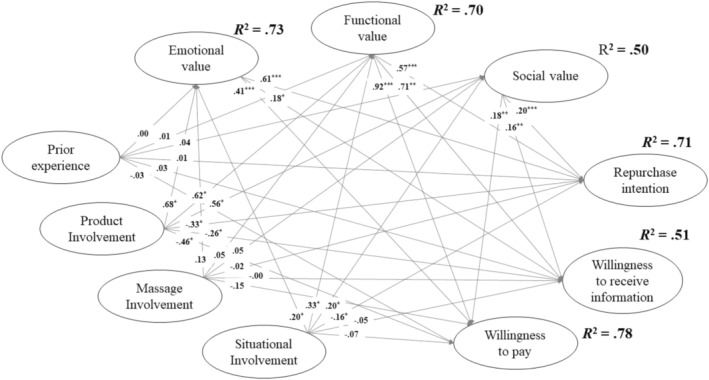
Results of SEM (*N* = 920). *p < 0.05, **p < 0.01, ***p < 0.001.

For consumer loyalty, the hypothetical mediation model achieved a good fit (*χ* ^2^ = 2141.515, df = 590, *χ* ^2^/df = 3.63, *p* < 0.001, RMSEA = 0.05, SRMR = 0.05, CFI = 0.91, and TLI = 0.90). With regard to repurchase intention, Figure 2 and Table 4 reveal that product and situational involvement had negative direct effects, whereas all three types of perceived value had positive direct effects. In terms of total effects, emotional value exhibited the strongest effect, followed by functional value, product involvement, social value, and situational involvement. The amount of variance explained reached 0.71.

**TABLE 4:** Effects of latent constructs on consumer loyalty (*N* = 920).

Independent & mediating constructs	Direct effects	Indirect effects	Total effects
**Dependent construct**	Repurchase intention		
Prior experience	0.01	0.01	0.02
Product involvement	−0.33*	0.88*	0.55**
Message involvement	−0.02	0.11	0.09
Situational involvement	−0.16*	0.35*	0.19*
Functional value	0.57***	—	0.57***
Emotional value	0.61***	—	0.61***
Social value	0.20***	—	0.20***
**Dependent construct**	Willingness to receive information		
Prior experience	0.03	0.01	0.04
Product involvement	−0.26*	0.65*	0.38**
Message involvement	−0.00	0.07	0.06
Situational involvement	−0.05	0.30*	0.25*
Functional value	0.71***	—	0.71***
Emotional value	0.18*	—	0.18*
Social value	0.16**	—	0.16**
**Dependent construct**	Willingness to pay		
Prior experience	−0.03	0.02	−0.01
Product involvement	−0.46*	0.94*	0.49**
Message involvement	−0.15	0.10	−0.05
Situational involvement	−0.07	0.42*	0.35**
Functional value	0.92***	—	0.92***
Emotional value	0.41***	—	0.41***
Social value	0.18**	—	0.18**

*Note:* *p < 0.05, **p < 0.01, ***p < 0.001.

With regard to willingness to receive information, the results reveal that product involvement had a negative direct effect, whereas all three types of perceived value had positive direct effects. In terms of total effects, functional value had the strongest effect, followed by product involvement, situational involvement, emotional value, and social value. The amount of variance explained reached 0.51.

With regard to willingness to pay, the results reveal that product involvement had a negative direct effect, whereas all three types of perceived value had positive direct effects; these results are similar to those obtained for willingness to receive information. In terms of total effects, functional value had the strongest effect, followed by product involvement, emotional value, situational involvement, and social value. The amount of variance explained reached 0.78.

### 4.5 Difference Analysis

An independent samples *t* test was conducted to assess the differences induced by sex and place of residence. The results revealed that men exhibited a significantly higher willingness to pay than women (Table 5); the remaining results did not exhibit significant differences between respondents with different sexes and places of residence.

**TABLE 5:** Results of the independent samples *t* test for customer loyalty (*N* = 920).

Construct	Men (*N* = 334)		Women (*N* = 586)		*t*	Levene	df
	*M*	SD	*M*	SD			
Willingness to pay	3.84	0.95	3.69	0.90	**2.51** *	2.06	918

*Note:* *p < 0.05.

Analysis of variance (ANOVA) was performed to evaluate the effects of age, educational level, monthly disposable income, and skin type on customer loyalty. The results indicated that respondents with a postgraduate degree had significantly lower repurchase intention and willingness to receive information than those without such a degree (Table 6). The other results did not exhibit significant differences between respondents with different ages, educational levels, monthly disposable incomes, and skin types.

**TABLE 6:** Results of the ANOVA for customer loyalty (*N* = 920).

Construct	High school and under (*N* = 116) (a)		Undergraduate (*N* = 546) (b)		Postgraduate (*N* = 258) (c)		*F*	Levene	df	Scheffé
	*M*	SD	*M*	SD	*M*	SD				
Repurchase intention	4.38	0.78	4.28	0.85	4.05	0.87	8.92***	1.05	2	a, b > c
Willingness to receive information	4.03	0.94	3.96	0.94	3.73	1.02	6.23**	1.33	2	a, b > c

*Note:* **p < 0.01, ***p < 0.001.

Table 7 reports the test results for the hypotheses proposed in this paper.

**TABLE 7:** Results of hypothesis tests.

Hypothesis	Results
H1: Perceived value positively affects customer loyalty.	S
H2: Prior experiences positively affect perceived value.	R
H3: Prior experiences positively affect customer loyalty.	R
H4: Perceived value enhances the effect of prior experiences on customer loyalty.	R
H5: Customer involvement positively affects perceived value.	PS
H6: Customer involvement positively affects customer loyalty.	PS
H7: Perceived value enhances the effect of involvements on customer loyalty.	PS
H8: For consumers of Taiwanese PEBSC products, customer loyalty is influenced by factors such as sex, age, place of residence, education level, monthly disposable income, and skin type.	PS

*Note:* PS refers to partially supported; R refers to rejected; S refers to supported.

## 5 Discussion

### 5.1 Constructs and SEM

In the factor analysis, prior experience refers to skin‐care products causing allergies or not meeting expectations. Product involvement includes respondent beliefs that Taiwanese PEBSC products are gentle, nonirritating, moisturizing, beautifying, and suitable for sensitive skin. Message involvement refers to respondent interest in product information such as target users, skin types, usage instructions, functions, and ingredient sources. Situational involvement refers to respondents' focus on product packaging design, store decoration style, and shopping website usability. In addition, functional value refers to the novelty, claimed functions, and benefits that the products offered to respondents. Emotional value refers to the comfort and trust that the products provide to respondents. Social value refers to the products' contribution to caring for the Earth and local agriculture and their environmental benefits. Repurchase intention refers to respondents' intention to continue purchasing the products. Willingness to receive information refers to respondent interest in receiving product information from skin‐care product manufacturers or friends and relatives. Finally, willingness to pay refers to respondent tolerance for price fluctuations in these products.

The results of SEM indicated that perceived value significantly influenced respondent loyalty to Taiwanese PEBSC products, thereby supporting the mediation model hypothesized in this study. Customer loyalty, particularly willingness to pay, was most strongly affected by functional value, followed by emotional value and social value. This finding suggests that marketing efforts for increasing customer loyalty toward Taiwanese PEBSC products should focus on providing customers with diverse and novel values.

### 5.2 Repurchase Intention

Emotional value had the strongest effect on repurchase intention, which indicates that emotional appeals to comfort and trust can profoundly enhance the repurchase rate of Taiwanese PEBSC products. Functional value and product involvement (mediated by perceived value) also had significant effects on repurchase intention. These constructs are related to usefulness, suggesting that the products should meet customers' expectations in the initial purchase. By contrast, social value and situational involvement had relatively weak effects on customer loyalty, which indicates that although environmental benefits, product packaging, and purchasing atmosphere have positive effects on repurchase motivation, they are not the key drivers of it. Prior experience and message involvement did not have significant effects on customer loyalty, even when the mediation by perceived value was considered. This result might be attributable to the fact that PEBSC products are still new in Taiwan and have a low market share; thus, advertisers might struggle to highlight their key selling points.

### 5.3 Willingness to Receive Information

Functional value had the strongest effect on willingness to receive information, which indicates that novelty and benefits should be the focus of product advertising. In addition, product involvement and situational involvement had a strong influence on willingness to receive information through perceived value, suggesting that functional descriptions that meet customer needs and an appealing presentation, including attractive packaging design and store decoration, can attract customers; the description should be prominently displayed in product advertising. Emotional value and social value had minor effects on willingness to receive information, which possibly indicates that appeals related to comfort, trust, and environmental benefits should not be the focus of product advertising. Message involvement and prior experience did not have significant effects on willingness to receive information. This result might be attributable to the fact that PEBSC products are not yet popular in Taiwan or that people who are already interested in these products do not need to receive the product information.

### 5.4 Willingness to Pay

Functional value had the strongest effect on willingness to pay, which suggests that novelty and benefits can drive premium purchasing behavior. Moreover, product involvement (mediated by perceived value) and emotional value had significant effects on willingness to pay, indicating that functions meeting customer needs and feelings of comfort and trust can encourage premium purchasing behavior. Situational involvement (mediated by perceived value) and social value had weak effects on willingness to pay, which possibly suggests that an appealing presentation, including attractive packaging design and store decoration, and environmental appeals do not significantly encourage premium purchasing behavior. Neither prior experience nor message involvement had a significant effect on willingness to pay, even when the mediation effect of perceived value was considered. This result might be attributable to the fact that PEBSC products are not yet widespread in Taiwan, and people who are familiar with such products might not engage in premium purchasing behavior.

### 5.5 Demographic Differences

Male respondents were more willing to pay a premium for PEBSC products than were female respondents. This result supports the conclusion of Duesterhaus et al. [68] that men believe high‐priced products symbolize high quality and low risk and consequently exhibit higher customer loyalty compared with women. Furthermore, respondents with a postgraduate degree were less likely to repurchase and receive information about the products than were those without such a degree. This result might be attributable to the fact that highly educated consumers are more budget‐conscious than less educated consumers, obtain information in diverse and rapid ways, and tend to believe that advertising content is biased [8].

## 6 Contributions and Suggestions

This study contributes to the fields of cosmetics, agriculture, and marketing in several ways. First, Taiwanese PEBSC products were identified as a promising avenue for development by considering global trends, and the causes of customer loyalty were deconstructed. This unique approach lays a solid foundation for future research. Second, a prediction model mediated by customers' perceived values was constructed as a novel framework for advancing academic understanding of customer loyalty toward skin‐care products. This model also serves as an empirical platform that can adapt to changes in plants and demographic variables in the future. Third, given Taiwan's considerable agricultural polarization, advanced food processing techniques, and external political threats, this study highlights the effects of local people's involvement on perceived value and customer loyalty for PEBSC products. The insights from this study can guide the development of relevant domestic policies, regulations, measures, and strategies for the agricultural and cosmetic industries.

Three recommendations are proposed for the Taiwanese government on the basis of the findings of this study. First, to ensure that Taiwanese PEBSC products remain innovative and diverse, companies should use a wider range of botanical ingredients. These ingredients, which can be derived from agricultural by‐products such as peels, seeds, and leaves, can be used to extract bioactive substances, fibers, and enzymes for product manufacturing. The government should continue to invest in the industrialization of agricultural science and technology, strengthen research and development for emerging ingredients, and enhance related production processes. Second, the safety of these skin‐care products can be addressed from two perspectives: raw materials and manufacturing processes. The presence of pesticide residues on crops is a common agricultural problem. Guiding farmers to use pesticides economically, rationally, and safely is a critical task for the government. Quality and hygiene depend on process management. Therefore, promoting compliance with best‐practice industrial standards for cosmetics at manufacturing sites can effectively improve process management and ensure consumer safety. Third, consumers view claims that a product is organic and natural as indicators of environmental friendliness, which can increase their willingness to purchase and pay a premium. To prevent false claims from misleading consumers, the government must enforce suitable regulations for organic and natural claims to protect consumers and legitimate businesses.

Moreover, two strategies for domestic agriculture are suggested. First, local farmers can collaborate to inventory their agricultural by‐products (including goodwill products) that require appropriate disposal. They can partner with nearby agricultural research institutes to identify items and ingredients with high extraction value. Through systematic coordination with local cooperatives, marketable products (such as skin‐care products or healthy foods) can be jointly developed. Second, farmers must understand that the circular economy is an effective strategy for preventing waste and reusing or recycling natural resources and agricultural by‐products. They should also focus on the rational and safe use of pesticides as a foundation for promoting the circular economy because pesticide residues can lead to unsellable agricultural products, cause safety issues, and affect the subsequent value‐added applications of agricultural by‐products.

Four strategies are also suggested for the cosmetic industry. First, when marketing Taiwanese PEBSC products, companies can highlight aspects such as gentleness, nonirritating nature, moisturizing ability, skin‐beautifying ability, and antiaging effect of these products to attract consumer attention. Emphasizing the novelty, diversity, and environmental friendliness of the natural, safe plant‐extracted ingredients can further enhance perceived value. Second, although consumers consider applicable targets, skin types, usage instructions, functions, and ingredient sources, establishing a positive user experience can be more impactful and credible than simply providing information. Experiential marketing is recommended to engage target audiences more effectively and increase their interest in the product information. Third, visual presentation is crucial for piquing people's interest. Excellent design can enhance a product's value. Companies can influence consumer perceptions regarding products directly and quickly through suitable packaging design or store decoration. Fourth, different marketing plans should be developed for different target groups. For example, high‐priced, high‐quality Taiwanese PEBSC products can be developed for men, appealing packages with diverse products can be developed for women, and authoritative product information can be used to win the trust of highly educated consumers.

This study has three limitations. First, skin‐care products in different categories, such as essences, moisturizers, hand creams, sunscreens, and face masks have different functions. Consumption behavior may vary depending on product attributes. However, this study did not conduct a detailed classification of product attributes, which might have biased the results. Second, only two questionnaire items evaluated prior experience, which might be insufficient for comprehensively measuring this construct. In addition, other variables affecting customer loyalty for PEBSC products can be investigated in future research. Third, sex significantly affects premium consumption, and education level significantly affects purchase intention and willingness to receive information. These results indicate that moderating effects might exist in the developed research model.

Future research should consider the following directions to address the aforementioned limitations. First, when consumer behavior research is conducted regarding Taiwanese PEBSC products, product type can be investigated in more detail. Moreover, specific plants (or plant families) can be studied separately, thereby reducing the potential bias caused in the results by differences in plant attributes. Second, prior experience can include various aspects, such as beauty, emotion, enjoyment, and relationship. Relevant questionnaire items can be developed to gain a deeper understanding of the relationship between prior experience and customer loyalty. Additional variables from different academic theories can also be included to supplement the results of the present study. Third, sex and education level can be considered moderating variables and included in the SEM model to gain a deeper understanding of the effects of demographic characteristics on consumption behavior.

## Consent

The authors confirm that they obtained oral informed consent from participants.

## Conflicts of Interest

The authors declare no conflicts of interest.

## Data Availability

Data are available on request from the authors.
